# Cryptochromes Mediate Intrinsic Photomechanical Transduction in Avian Iris and Somatic Striated Muscle

**DOI:** 10.3389/fphys.2020.00128

**Published:** 2020-02-21

**Authors:** Joseph F. Margiotta, Marthe J. Howard

**Affiliations:** Department of Neurosciences, University of Toledo College of Medicine and Life Sciences, Toledo, OH, United States

**Keywords:** eye, iris, striated, muscle, light, cryptochrome, photomechanical, circadian

## Abstract

Irises isolated from the eyes of diverse species constrict when exposed to light. Depending on species this intrinsic photomechanical transduction response (PMTR) requires either melanopsin or cryptochrome (CRY) photopigment proteins, generated by their respective association with retinoid or flavin adenine dinucleotide (FAD) chromophores. Although developmentally relevant circadian rhythms are also synchronized and reset by these same proteins, the cell type, mechanism, and specificity of photomechanical transduction (PMT) and its relationship to circadian processes remain poorly understood. Here we show that PMTRs consistent with CRY activation by 430 nm blue light occur in developing chicken iris striated muscle, identify relevant mechanisms, and demonstrate that similar PMTRs occur in striated iris and pectoral muscle fibers, prevented in both cases by knocking down *CRY* gene transcript levels. Supporting CRY activation, iris PMTRs were reduced by inhibiting flavin reductase, but unaffected by melanopsin antagonism. The largest iris PMTRs paralleled the developmental predominance of striated over smooth muscle fibers, and shared their requirement for extracellular Ca^2+^ influx and release of intracellular Ca^2+^. Photo-stimulation of identified striated myotubes maintained in dissociated culture revealed the cellular and molecular bases of PMT. Myotubes in iris cell cultures responded to 435 nm light with increased intracellular Ca^2+^ and contractions, mimicking iris PMTRs and their spectral sensitivity. Interestingly PMTRs featuring contractions and requiring extracellular Ca^2+^ influx and release of intracellular Ca^2+^ were also displayed by striated myotubes derived from pectoral muscle. Consistent with these findings, cytosolic CRY1 and CRY2 proteins were detected in both iris and pectoral myotubes, and knocking down myotube *CRY1/CRY2* gene transcript levels specifically blocked PMTRs in both cases. Thus CRY-mediated PMT is not unique to iris, but instead reflects a more general feature of developing striated muscle fibers. Because CRYs are core timing components of circadian clocks and CRY2 is critical for circadian regulation of myogenic differentiation CRY-mediated PMT may interact with cell autonomous clocks to influence the progression of striated muscle development.

## Introduction

Irises isolated from the eyes of fish, amphibians, reptiles, birds as well as nocturnal and crepuscular mammals constrict within seconds when exposed to light (reviewed by Barr, 1989; Xue et al., 2011). This intrinsic photomechanical transduction response (PMTR) is postulated to be initiated by a “light sensor” associated with the iris sphincter muscle that triggers an increase in free intracellular Ca^2+^ (Zucker and Nolte, 1978; Barr, 1989; Tu, 2004; Xue et al., 2011). Melanopsin (OPN4) and Cryptochrome (CRY) are photopigments expressed in irises that underlie PMT in a species-dependent manner; they also function over longer times to exert neural control over circadian rhythms and cell autonomous circadian clocks. OPN4 induces phototransduction in retinal ganglion cells which transmit day/night cycle information to the hypothalamic suprachiasmatic nucleus (SCN) a “master pacemaker” that generates rhythmic output firing allowing it to synchronize peripheral circadian clocks through neural and humoral pathways (Paul et al., 2009; Welsh et al., 2010). Correlative and gene knockdown studies indicate that OPN4 underlies the PMTR in *Xenopus* iris (OPN4X) and the mammalian form (OPN4M) is at least partly responsible for the PMTR in mouse iris muscle, acting *via* phospholipase-C (PLC) to trigger an increase in intracellular Ca^2+^ (Provencio et al., 1998; Lucas et al., 2003; Xue et al., 2011; Wang et al., 2017). Unlike the OPN4s, that utilize retinoid based light-sensitive chromophore, Type I CRYs are classified as flavoproteins in that they stoichiometrically bind a flavin adenine dinucleotide (FAD) chromophore to initiate a phototransduction process (Oztürk et al., 2008). Type I CRYs signal *via* blue light FAD photoreduction/phosphorylation, and are distantly related to photolyases responsible for repairing DNA damaged by ultraviolet light. *Drosophila* Type I CRY is instrumental in light-dependent magnetoreception (Gegear et al., 2008) and phototransduction in arousal neurons (Fogle et al., 2011, 2015) and also has transcriptional activity, transducing light-dependent molecular interactions important for circadian clock functions (Busza et al., 2004). In vertebrates, CRYs are molecular components of circadian clocks that drive light-independent expression of cell-specific genes, and have been classified as Type II. In mammals CRYs set clock periodicity by acting as transcriptional repressors in a negative feedback cycle, dimerizing with Per proteins in the cytoplasm and translocating to the nucleus where they inhibit CLOCK:BMAL1 factors from initiating further transcription (Griffin et al., 1999; Cashmore, 2003; Mohawk et al., 2012; Buhr and Takahashi, 2013).

Avian CRYs pose an exception to the simple invertebrate light-dependent (Type I) *versus* vertebrate light-independent (Type II) classification since an avian CRY (CRY4) binds FAD and plays a light-dependent role in magnetoreception (Kubo et al., 2006; Watari et al., 2012; Wang et al., 2018). Moreover, avian CRY1 and CRY2 share 14 of the 17 amino acids with CRY4 at sites implicated in FAD binding as well as a triad of tryptophans believed to facilitate light-activated intramolecular electron transfer (Kubo et al., 2006). Consistent with a role in PMT, the robust PMTR observed in embryonic chicken iris (reviewed by Pilar et al., 1987) requires CRY1 and CRY2. Specifically, PMT persisted after retinoid depletion, its action spectrum with a peak at 430 nm matched that for bacterial CRY absorption but not that of any opsin-based photopigment, and PMTRs were attenuated after siRNA *CRY1* and *CRY2* transcript knockdown but persisted after *OPN4* knockdown (Tu, 2004). Despite strong evidence for CRYs underlying chick iris PMT, the relevant light sensing cells have not been identified and neither their dependence on CRY proteins therein, nor relevant downstream signaling mechanisms have been identified. To approach these gaps, cellular and molecular mechanisms underlying PMT in chick iris and muscle fibers were explored. Here we show that PMTRs consistent with CRY activation occur in developing chick iris striated muscle, identify relevant mechanisms, and demonstrate that similar PMTRs occur in striated iris and pectoral muscle fibers that are prevented in both cases by knocking down *CRY1*and *CRY2* gene transcripts with anti-sense oligonucleotides. The results indicate that cytosolic CRY-photoactivation couples to mobilization of intracellular Ca^2+^, mediating PMT and reflecting a shared feature of developing striated iris and somatic muscle.

## Materials and Methods

### Chick Iris Preparation

According to PHS policy, no vertebrate animals will be used. “PHS Policy is applicable to proposed activities that involve live vertebrate animals. While embryonal stages of avian species develop vertebrae at a stage in their development prior to hatching, OPRR has interpreted “live vertebrate animal” to apply to avians (e.g. chick embryos) only after hatching^1^.”. Fertile chicken (*Gallus gallus*) eggs were obtained from the Department of Animal Sciences, Michigan State University and housed in a forced draft egg incubator at 38°C. After 8–18 days of embryonic development (E8-18) (Hamburger and Hamilton, 1951) iris preparations were excised from the eyes of embryos of both sexes by making circumferential cuts along the cornea-scleral border and gently pulling the resulting disk free from the underlying lens and vitreous humor. The preparations, including iris sphincter muscle as well as associated dilator, pigment epithelium (PE) and cornea and were pinned cornea down, maintained at 21–22°C in a recording solution (RS; pH 7.4) containing (in mM): 145 NaCl, 5.3 KCl, 5 HEPES, 5.4 Glucose, 0.8 MgSO_4_, and 2.5 CaCl_2_, and dark adapted for 15–20 min between photo-stimulation trials. In some cases, the PE was removed with a cotton-tipped applicator.

### Cell Culture

Because the PMT light sensor is postulated to be within the iris sphincter muscle (Barr, 1989; Tu, 2004; Xue et al., 2011) a culture system was employed to assess its cellular basis. Cultures were prepared from 6 to 12 E13-14 iris sphincter muscles and their associated PE, or from 2 to 3 E11-12 pectoral muscles. Dissociated cells were obtained by pre-incubating dissected tissue pieces for 20–30 min at 37°C followed by trituration and filtration in growth medium. The growth medium consisted of Eagle’s minimum essential medium (MEM) supplemented with 2 mM glutamine, 100 U/ml penicillin, 100 μg/ml streptomycin, 10% heat-inactivated horse serum (all components from Invitrogen) and freshly-prepared 5% E11 chick embryo extract (MEM^HS/CEE^). For Ca^2+^ imaging, triturated samples were plated in MEM^HS/CEE^ on 12 mm diameter glass coverslips or on 35 mm glass-bottom WillCo-dishes (BioSoft International) that were pre-coated with 100-300 KdA poly-d-lysine hydrobromide (1 mg/ml, Sigma P1149) and Collagen (0.1%, Gibco A-10483). For RNA extraction, triturated samples were plated at high-density (>100 × 10^3^/ml) in identically pre-coated 25 mm diameter plastic tissue culture wells. Cultures were maintained in 95% air/5% CO_2_ at 37°C for 4–7 days and MEM^HS/CEE^ was replenished every 2–3 days. In cultures used for RNA extraction MEM^HS/CEE^ was supplemented cytosine arabinoside (ARA-C, 5–10 μM) added 2–3 days after plating to inhibit proliferation of rapidly dividing non-muscle cells.

### Whole Iris Photostimulation and Data Analysis

Light was applied to iris preparations from a Xenon source (Zeiss 75W) filtered through 360 nm longpass and 550 nm shortpass filters (ET360lp, #NC548428 Chroma and Techspec SP, #84-708 Edmund Optics). The resulting broadband 360 to 550 nm violet-blue-cyan light was focused through a 30 mm condenser lens and applied *via* a 1.5 mm core optical fiber cable (Condenser ACL3026-A, and cable 1500UMT 0.39 NA; Thorlabs). Light power was measured using a photodiode sensor (200–1100 nm, 50 mW, 9.5 mm diameter) and optical power meter (Thorlabs S120VC and PM100D) and had a median irradiance value of 25 mW cm^–2^ for the 360 to 550 nm filtered light, similar to that used previously to evoke the PMTR in chick iris (35 mW cm^–2^, for 350 to 590 nm light) (Tu, 2004). For controls, 525 nm orange light (25–35 mW cm^–2^) was applied using a longpass filter (#84-744 Edmund Optics). Preparations were viewed at 12X with a Wild dissecting microscope and digital images acquired (usually at 1Hz for 25–60 s) using a cooled CCD camera (Retiga 1434, Q-Imaging) controlled by IP Lab 4.0 (Scanalytics Software; Reading PA). PMTRs were quantified offline from iris sphincter open areas measured at each time point using macros written for Image J (v 1.45s). Iris responses to pharmacological stimulation were obtained in red light, but acquired and analyzed in a similar fashion.

### Myotube Photosensitivity, Photostimulation and Data Analysis

Myotube photosensitivity was assessed by imaging changes in [Ca^2+^]_in_ using the Ca^2+^ indicator Rhod-2 (Molecular Probes, 80% maximal excitation at 540 nm). After 4–6 days myotube cultures were loaded with 2–5 μM Rhod-2-AM in RS at 22°C for 30–45 min, washed, dark-adapted for 15–20 min and examined in RS using a Zeiss 200M inverted microscope equipped with a HBO 100 Mercury Arc light source and a Zeiss Plan-Neofluar 40X/0.75NA objective. To achieve simultaneous, non-overlapping PMTR activation and Rhod-2 excitation/emission in myotubes, light was applied *via* a dual wavelength excitation filter (435/10x nm and 535/10x nm excitation; Chroma 59033X). The dual wavelength light was attenuated using a 1.0 OD neutral density (ND) filter and applied to myotubes *via* the 40X objective. In a previous report, peak PMTRs were achieved in chick iris using 430 nm light delivered at irradiances of 7–14 mW cm^–2^ (corresponding to photon flux values of 1.5–3.0 × 10^16^ photons s^–1^cm^–2^) and undetectable using 530 to 550 nm light at the same fluxes (Tu, 2004). After measuring the excitation irradiance and integrating the respective power outputs from the HBO light source applied through the 1.0 OD ND filter and the 40X objective, calculated photon flux values at the 435 and 535 nm wavelengths were similar (1.5 × 10^16^ and 1.8 × 10^16^ photons s^–1^ cm^–2^, respectively) with the 435 nm flux sufficient to activate PMTRs, and the 535 nm flux exciting Rhod-2 but causing minimal or no PMTR (Tu, 2004). In control experiments, a 545 nm single wavelength CY3/TRITC excitation filter (ET545/25x; Chroma 49004 ET) and 1.0 OD ND filter were used to deliver 545 nm light to Rhod-2 loaded myotubes at ≈3 × 10^16^ photons s^–1^ cm^–2^.

Light was applied to fields containing segments of 1–3 myotubes for 25–90 s and time-lapse images were simultaneously acquired at 1–2 Hz for exposure times of 50–200 ms. Camera, exposure, and light stimuli timing were controlled by IP Lab 4.0 (Scanalytics Software; Reading, PA). Changes in [Ca^2+^]_in_ were assessed off-line from the acquired image stacks using custom macros written for Image-J (v 1.45s) to determine at each time the average fluorescence intensity within a region of interest (ROI) circumscribing the myotube perimeter (F_t_) and the associated average background fluorescence intensity (F_B,t_). For each time point ΔF (F_t_ − F_B,t_) was normalized to F_B,t_ to obtain the net fluorescence intensity relative to background (ΔF/F_B_). In some cases, light induced concentric myotube contractions were assessed in the same image stacks by measuring the distance between two fluorescent spots (usually nuclei) along the longitudinal axis of the resting and contracted myotube.

In control experiments Ca^2+^ responses were induced in Rhod-2 loaded myotubes by AChR activation or membrane depolarization. To do so RS containing carbachol (CCh, 1 mM) or KCl (100 mM) was applied from blunt patch pipettes by pressure microperfusion (5 psi, 10 s) and myotube Ca^2+^ responses assessed using single wavelength 545 nm light.

### Myotube nAChR and CRY Detection

nAChRs were detected by labeling live myotube cultures with Alexa Fluor 488 conjugated α-Bungarotoxin (AF488-αBgt, Molecular Probes). Coverslip cultures were equilibrated to room temperature (RT, 21°C) washed 3X in 0.1 M phosphate buffered saline (PBS) containing BSA (2 mg/ml), incubated with AF488-αBgt (1:250), followed by PBS washing, fixation in paraformaldehyde (2–4% in PBS, 20 min), PBS washing, and mounting (Fluoromount-G, Invitrogen) on glass slides. CRY proteins were detected by immunolabeling muscle cultures with anti-CRY1 or anti-CRY2 polyclonal antibodies (pAbs; Boster, PB9540 or PB9576, respectively) raised against synthetic N-terminal human CRY peptides. The human CRY peptide immunogens (CRY1: F_153_QTLISKMEPLEIPVETITSEVIEKCTTPLSDDHDEK_189_ and CRY2: R_171_FQAIISRMELPKKPVGLVTSQQMESCRAE_200_) were 89 and 90% identical, respectively, to corresponding regions of *Gallus* CRY1 and CRY2. Coverslip cultures were equilibrated to RT, washed 3X in 0.1 M PBS, fixed in paraformaldehyde (2–4% in PBS, 20 min), permeablized in block solution (BS; 0.1 M Tris, 0.3% Triton X-100, 5% donkey serum), incubated in BS containing CRY1 or CRY2 pAb (5–10 μg/ml; 16 h, 4°C), washed, treated with AF546-conjugated donkey anti-rabbit IgG secondary antibody (4 μg/ml in BS, 1 h, RT) washed, in some cases treated with the nuclear marker 4′,6-diamidino-2-phenylindole (DAPI, 300 nM, 5 min) washed 3X in PBS and mounted as above. Labeled cultures were examined with epifluorescence optics using the same Zeiss 200M inverted microscope, and 40X objective as for photosensitivity measurements with filter sets appropriate for AF488 or AF546 detection (480/30 nm excitation, 535/40 nm emission Chroma 31001, or 545/25 nm excitation, 605/70 nm emission Chroma 49004 ET). To assess CRY localization in cytoplasm and nuclei, CRY/DAPI co-labeled cultures were examined using a Leica TCS SP5 laser scanning confocal microscope (Leica Microsystems, Bannockburn, IL, United States) equipped with conventional solid state and a Ti-sapphire tunable multi-photon laser (Coherent, Santa Clara, CA, United States). Images were acquired at 512 × 512 pixels with a 63X objective (NA 1.40) in 1 μm thick optical sections. Images were collected using a motorized galvanometer in sequential scan mode with laser power, gain and offset optimized to minimize saturation and avoid background. Nuclear DAPI labeling was excited with the multiphoton laser at 790 nm and emission at 400–483 nm, while AF546 CRY labeling was excited with the conventional laser at 488 nm (to minimize photobleaching) and emission detected at 554–605 nm.

### Photopigment Transcript Detection and CRY Knockdown

Transcripts encoding chicken (*Gallus gallus*) melanopsin (OPN4) and CRY subtypes (*OPN4M*, *OPN4X*, *CRY1*, and *CRY2*) and the housekeeping protein, glyceraldehyde 3-phosphate dehydrogenase (*GAPDH*) were detected in E11 and 17 iris, E14 pectoral muscle, and (for *CRY1, CRY2 and CRY4*) in 4–7 days pectoral muscle cultures by reverse transcription-based polymerase chain reaction (RT-PCR). Total RNA was isolated from iris and pectoral muscle and cultures (RNAqueous-Micro kit, Ambion), treated with 0.1 U/μl DNase 1 (New England Biolabs) and quantified by spectrophotometry (NanoDrop 1000, Thermo Scientific). RNA was converted to cDNA in RT reactions containing 2.5 U/μl MultiScribe reverse transcriptase and 2.0 U/μl RNAse inhibitor (2 h at 37°C, followed by 5 min at 85°C, both enzymes from Applied Biosystems). The cDNA templates were amplified in reactions containing 0.08 U/μl Platinum Taq DNA Polymerase (Invitrogen), MgCl_2_ and relevant primer pairs listed below (0.05–0.10 μM for *GAPDH* set 1, 0.1–0.2 μM for *OPN4X* and *OPN4M*, and 0.1–0.2 μM for *CRYs*). The PCR products and DNA molecular weight markers (pGEM 75–2645 bp; Promega) were loaded on 1.5% agarose gels containing EtBr and separated by electrophoresis (70 V, 2 h). Images of EtBr stained products were visualized with UV light, acquired with a digital CCD camera (Kodak DC290), and bands identified using Kodak 1D software.

*Gallus GAPDH* (NM_204305.1)

Primer Set 1:
Forward: 5′-G_589_CCATCACAGCCACACAGAA_608_-3′
Reverse: 5′-A_1037_CCATCAAGTCCACAACACG_1018_-3′
PCR product Size: 449 bp

Primer Set 2:
Forward: 5′-C_265_ACGCCATCACTATCTTCCAGGA G_288_-3′
Reverse: 5′-C_786_AGGTCAACAACAGAGACATTGG G_763_-3′
PCR product Size: 522 bp

*Gallus CRY1* (NM_204245.1)

Primer Set (Tu, 2004):
Forward: 5′-A_1440_ATGCCCCAGAGAGTGTCCAGAAG_1463_-3′
Reverse: 5′-C_1740_ACATGTCTGAACGCCAACTGTC_1718_ -3′
PCR product Size: 301 bp

*Gallus CRY2* (NM_204244.1)

Primer Set (Tu, 2004):
Forward: 5′-G_1399_CCAAGTGCATCATTGGAGTGG_1420_ -3′
Reverse: 5′-C_1684_TTCAGTGCACAGCTCTTCTGCTC_1661_-3′
PCR product Size: 286 bp

*Gallus CRY4* (XM_015298682.2)

Primer Set (Based on Wang et al., 2018)
Forward: 5′-G_1801_AGGAGGGGAGAGCGAAGG_1819_-3′
Reverse: 5′-A_1963_AGCTGCGGACTGACAGGCA_1944_-3′
PCR product Size: 163 bp

*Gallus OPN4m* (NM_001044653.1)

Primer Set (Verra et al., 2011):
Forward: 5′-T_1710_CTCGCCGTAGAACATCC_1727_-3′
Reverse: 5′-G_1954_AAGTGTTTCAGAGCAAGGTAGGA_1931_-3′
PCR product Size: 245 bp

*Gallus OPN4x* (GenBank AY036061.1)

Primer Set (Chaurasia et al., 2005; Verra et al., 2011):
Forward: 5′-T_235_GCTTTGTCAACAGCTTGCACAGAG_259_-3′
Reverse: 5′-C_433_AGCAATAATCTGTATGGTGCGCTTC_408_-3′
PCR product Size: 199 bp

To achieve *CRY* knockdown, three *CRY1* and three *CRY2* antisense oligonucleotides (ASOs) with 2′-deoxy-2′-fluoro-beta-D-arabinose sugar (FANA) modifications were synthesized (21 nt each, AUM BioTech) and assessed for their ability to knock down *CRY1* or *CRY2* transcripts. In this case 2–3 days pectoral muscle cultures were switched to MEM^HS/CEE^ containing 5–10 μM ARA-C, with or without 2–5 μM *CRY1* or *CRY2* ASO for 24–48 h, and RNA isolation and RT conducted as above. For quantification, PCR conditions were optimized to ensure sub-saturating product amplification of both *GAPDH* and *CRY* products in the same reactions (Marone et al., 2001). Optimization involved using cDNA templates derived from 100 to 150 ng RNA, using *GAPDH* primers (set 2) at 0.1 μM, and MgCl_2_ at 2 mM, and adjusting thermocycler parameters for CRY1 and CRY2 to 5 min at 95°C followed by 24–26 cycles of 45 s at 95°C, 45 s at 65°C and 1 min at 72°C, and for CRY4 to 3 min at 95°C followed by 30 cycles of 60 s at 95°C, 75 s at 68°C and 45 s at 72°C. Knockdown efficiency (E_KD_) was evaluated using Kodak 1D analysis software by comparing EtBR-fluorescence intensity in ROIs drawn around *CRY* product bands (F_C,ROI_) minus background intensity from an equivalent ROI (F_B_) divided by F_B_ (ΔF_C_/F_B_ = (F_C,ROI_−F_B_)/F_B_) relative to a similarly calculated value for the *GAPDH* product band (ΔF_G_/F_B_). To determine the effect of *CRY* knockdown on PMT in myotubes from iris or pectoral muscle cultures, MEM^HS/CEE^ containing *CRY1 and CRY2* ASOs that maximally reduced transcripts, or a scrambled oligonucleotide (2–5 μM each) were applied to 4–5 days iris or pectoral muscle cultures for 24–48 h. Oligonucleotide-treated and control cultures were then washed, loaded with Rhod-2, and myotubes assayed for PMTRs using dual excitation 435 and 535 nm wavelength light stimulation as described above. We assume that ASO efficacy is unaffected by the minor differences in culture conditions employed for RNA *versus* functional analyses. In control experiments, single excitation 545 nm wavelength light stimulation was used to compare Rhod-2 Ca^2+^ responses to membrane depolarization (achieved by focal application of 100 mM KCl) in control and ASO-treated muscle fibers.

### Statistics

Parameter values are expressed as mean ± SEM, followed by the number of irises or myotubes tested (*n*) and, for myotube cultures by the number (*N*) of individually cultured coverslips. Values obtained following drug treatments are presented relative to those obtained for sham-treated controls from the same day or culture (1.00) such that a fold-change value of 0.5 indicates a 50% decrease. Statistical comparisons were made using Prism software (v 5.0d GraphPad, La Jolla, CA, United States). Statistical significance was assessed using Students unpaired, two-tailed *t*-test with criterion cutoff at *p* < 0.05 following Welch’s correction for unequal variances, when necessary. Asterisks (^∗^) in figures indicate a significant difference (assessed by *p* < 0.05) between *n* values for test and control measurements.

## Results

### Iris PMT Is Generated by Striated Muscle

**FIGURE 1:**
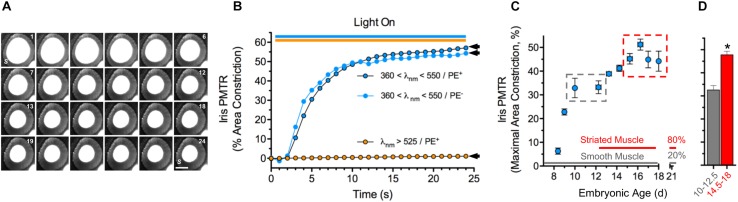
Chick iris intrinsic *PMT*. **(A)** Typical PMTR images (E16 chick iris preparation). Broadband filtered violet-blue-cyan light (360 < λ < 550 nm, 25 mW cm^–2^) applied *via* a fiber optic light pipe (lower right) was switched on at 1 (sec), off at 24 (top right). Note that the light induced a PMTR in the iris sphincter muscle (*S*) causing constriction of the pupil (white center bounded by sphincter) reducing its area from 3.3 mm^2^ at onset to 1.4 mm^2^ at 24 s. *S* = iris sphincter muscle. Scale bar = 1 mm. **(B)** Time course and spectral sensitivity of PMTR in E14-16 irises. Violet-blue-cyan light (blue circles) produced robust PMTRs that typically constricted pupil areas by ≈50% within 20 s, while orange light (λ > 525 nm, orange circles) at similar irradiance produced no response. Arrowheads indicate maximal area constrictions as presented in Figures 2, 3 which employed the same light stimulus parameters. Note that the presence of pigment epithelium (PE, black bordered symbols) did not affect the amplitude or kinetics of the PMTR. **(C)** Development. Iris PMTR, assessed as maximal iris constriction, increased in a stepwise fashion during the developmental period from E8.5 - E18. PMT was first detectable at E8.5 with maximal constriction increasing to a plateau at E10-12.5 and again to a second plateau at E14.5-18 (gray and red dashed boxes). **(D)** Maximal PMT values increased significantly between early (E10-12.5, gray column; *n* = 35) and later (E14.5-18, red column; *n* = 89) developmental periods. Note that the developmental increase in PMT plateau values was correlated with the appearance and eventual predominance of striated muscle fibers (80%) over smooth muscle fibers (20%) as previously described for the chick iris (Yamashita and Sohal, 1986). In this and subsequent figures asterisks (*) indicate a significant difference between *n* test and control measurement values (*p* < 0.05, Students’ two-tailed *t*-test).

Broadband violet-blue-cyan light (360 < λ < 550 nm @ 25 mW cm^–2^) induced PMTRs in embryonic day 14.5–18 (E14.5-18) chick irises, featuring ≈50% constriction of pupillary open area within 25 s (Figures 1A,B). While the iris preparations contain sphincter and dilator muscles as well as pigment epithelium (PE) PMTRs arose specifically from sphincter muscle because identical responses were obtained in preparations after removing the PE or when connections to the dilator were damaged (latter not shown). Consistent with previous action spectrum studies indicating PMTR specificity for 430 nm light (Tu, 2004) filtered orange light (λ > 525 nm, 25–35 mW cm^–2^) failed to evoke pupil constriction, and neither light stimulus paradigms detectably changed recording solution (RS) temperature. To explore the PMTR dependence on sphincter muscle fiber type, irises were isolated at developmental stages corresponding to the presence of smooth and striated muscle fibers, respectively (Yamashita and Sohal, 1986). The largest PMTRs occurred at stages when irises contain mostly striated muscle fibers (Figures 1C,D). Photomechanical responses to 360 < λ < 550 nm light were barely distinguishable in irises isolated at E8, then rose to plateau values of ≈30% constriction at E10-12.5, stages when only smooth muscle fibers are present (Yamashita and Sohal, 1986). After E13, PMTRs rose to a second plateau with 40–50% pupil constriction observed between E14.5-18, times associated with the appearance and predominance of striated over smooth muscle fibers (80 versus 20% by E21) (Yamashita and Sohal, 1986). Finding that chick iris PMT does not require PE and correlates with the developmental appearance and predominance of striated muscle supports the view that it is triggered by a light sensor in striated sphincter muscle. In addition, the requirement for 360 < λ < 550 nm light seen here at E14.5-18 is consistent with previous studies implicating CRYs as the photosensitive molecules underlying PMT in E15 irises (Tu, 2004).

**FIGURE 2:**
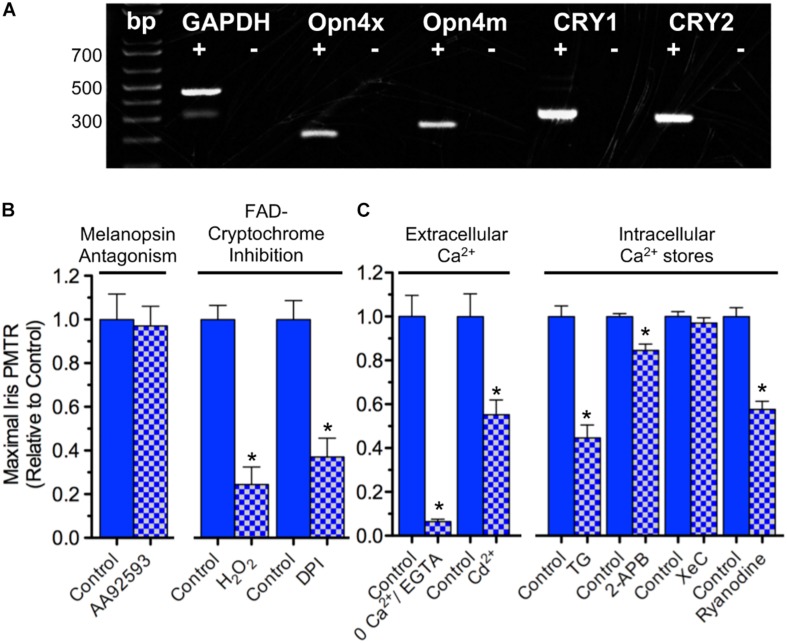
Iris PMT mechanism*.* **(A)** Expression of *OPN4* and *CRY* transcripts in the chick iris. PCR was conducted on cDNA templates obtained by reverse transcription of iris sphincter muscle RNA isolated at E17 (2 separate experiments) using primers based on chicken-specific sequences. OPN4x and OPN4m denote chick *Xenopus*- and mammalian-like melanopsin. CRY1 and CRY2 denote CRY subtypes. Reverse transcriptase presence or absence in the PCR reactions is indicated by + and – symbols, respectively. Reaction products were of predicted size (bp). **(B)** Involvement of CRY over OPN4 photopigments in PMT. (Left) Incubating irises with the small molecule OPN4 phototransduction inhibitor AA92593 (10–20 μM, 1–3 h) had no effect on iris PMT relative to time-matched controls (*n* = 4 test and control irises). (Right) Consistent with involvement of Flavin-containing, redox-signaling CRY proteins, inhibition of flavin-specific reduction following incubation with DPI (10 μM, 3 h) or increasing oxidation with H_2_O_2_ (0.4 mM, 1–2 h) significantly reduced PMT by 63 ± 6 or 76 ± 8%, respectively, relative to time-matched controls (*n* = 4 test and control irises in both cases). **(C)** Ca^2+^ dynamics underlying iris PMT. (Left) PMT requires external Ca^2+^ influx. PMTRs were reduced by 94 ± 1% in irises incubated for 10–20 min in Ca^2+^-free RS buffered with 2 mM EGTA (*n* = 3 test and 3 control irises) and reduced by 45 ± 7% in irises incubated for 30 min in RS (normal Ca^2+^) containing 2 mM Cd^2+^ to inhibit influx *via* Ca^2+^ channels (*n* = 8 test and 8 control irises). (Right) Iris PMT requires Ca^2+^ release from an intracellular store. Incubation in RS containing Thapsigargin (TG, 3 uM, 1.5h) to block the SERCA pump reduced the light response by 55 ± 6% (*n* = 5 test and 5 control irises). IP3-receptor inhibition with 2-APB (30–50 μM, 1.5 h) or with Xestospongin-C (XeC, 2 μM, 1.5 h) had little or no significant effect (*n* = 4–6 test and time- matched control irises). By contrast, RyR mediated Ca^2+^ release appeared critical since Ryanodine (100 μM, 1.5 h) significantly reduced the PMT by 42 ± 4% (*n* = 5 test and 5 time matched control irises). Results are expressed as mean maximal PMTR (±SEM) for irises in test RS conditions (blue/white check columns) relative to control irises (blue columns) from the same experiments assayed in normal RS.

Because mRNA transcripts encoding OPN4 and CRY proteins are expressed in chick iris (Figure 2A) pharmacological tests were performed to further assess their respective relevance to iris PMT. AA92593 is a small molecule inhibitor of *Homo sapiens* OPN4-mediated phototransduction that acts by competing with chromophore (i.e. retinoid) binding (Jones et al., 2013). AA92593 has not been tested as a melanopsin antagonist in other species, but it is expected to also recognize chicken (*Gallus*) OPN4s. Specifically, OPN4s from *Homo sapiens*, *Gallus* (and *Xenopus*) share 90% identity in transmembrane segment 7 (TM7) including a conserved Lys residue therein that acts as a site for Schiff base retinoid linkage (Wang et al., 1980) as well as 77% identity in TM3 including a conserved aromatic Tyr residue believed to stabilize retinoid binding (Provencio et al., 1998). Consistent with previous work showing that PMTRs persisted after either retinoid depletion or siRNA-mediated *OPN4* transcript knock down (Tu, 2004) AA92593 failed to reduce chick iris PMTRs (Figure 2B). By contrast, interfering with CRY-FAD signaling by inhibiting flavin-specific reduction with diphenyleneiodonium (DPI) or by simulating FAD oxidation with H_2_O_2_ significantly reduced PMTRs (Figure 2B). While redox interference can affect other cellular functions, DPI has been used previously to inhibit light-induced CRY-FAD signaling in *Drosophila* (Fogle et al., 2011) and reoxidation is thought to promote the CRY-associated FAD resting state (van Wilderen et al., 2015). These latter findings are also consistent with previous results, which showed that siRNA-mediated *CRY1* and *CRY2* transcript knockdown reduced PMTRs (Tu, 2004). Thus the pharmacological tests with AA92593, DPI and H_2_O_2_ provide additional evidence that, at developmental stages associated with striated sphincter muscle predominance, the chick iris PMTR requires CRYs whereas OPN4s appear dispensable.

Possible downstream targets were next probed to determine how CRYs generate PMT in chick iris. Na^+^ channel activation was not required since iris PMTRs were unaffected by incubation with tetrodotoxin (data not shown and Tu, 2004; Xue et al., 2011). Also dispensable were nicotinic and muscarinic acetylcholine receptors (nAChRs and mAChRs) that are present on striated iris sphincter muscle fibers and underlie nerve-evoked iris constriction (Pilar et al., 1987). Their possible activation (e.g. by light evoked ACh release from presynaptic terminals) was also not required for PMT because light-evoked iris area constrictions obtained during 15–30 min incubations in RS containing (ATR, 1 μM) and 100 μM d-tubocurarine (dTC, 100 μM), competitive inhibitors of the respective AChR classes, were indistinguishable from controls tested in parallel (47 ± 5%, *n* = 13 versus 52 ± 2%, *n* = 22). The ATR/dTC inhibitor cocktail was effective, however, in inhibiting AChRs; identical treatments reduced iris constrictions induced by perfusion with the pan-specific AChR agonist Carbachol (CCh; 1 mM) by 72 ± 6% relative to untreated controls (*n* = 6 each). Consistent with the importance of Ca^2+^ dynamics in muscle contraction (Kuo and Ehrlich, 2015) both extracellular Ca^2+^ influx and Ca^2+^ release from intracellular stores were involved in generating chick iris PMT (Figure 2C). Extracellular Ca^2+^ was required because light-evoked iris area constriction was reduced by >90% when irises were tested in extracellular RS containing 0 Ca and 2 mM EGTA. In *Drosophila* arousal neurons, CRY signaling couples to membrane depolarization leading to an increased rate of action potential firing (Fogle et al., 2011, 2015). Consistent with CRY signaling coupling to Ca^2+^ influx *via* membrane depolarization, light induced pupil constrictions were reduced by 45% in irises tested in RS containing Cd^2+^, a generic inhibitor of voltage- (i.e. depolarization-) gated Ca^2+^ channels (VGCC) (Swandulla and Armstrong, 1989). In skeletal muscle, VGCC mediated Ca^2+^ influx triggers Ca^2+^ release from intracellular sarcoplasmic reticulum (SR) stores, thereby allowing Ca^2+^ to activate the contractile apparatus (Endo, 2009; Kuo and Ehrlich, 2015). In accord with previous findings (Tu, 2004) chick iris PMT also requires Ca^2+^ release from intracellular sarco/endoplasmic stores because incubation in RS containing Thapsigargin (TG) to block the sarco/endoplasmic Ca^2+^-ATPase (SERCA) pump and deplete Ca^2+^, reduced PMTRs by 55%. Contrasting with the requirement for PLC and subsequent IP3-receptor mediated Ca^2+^ release implicated in mammalian smooth muscle iris (Xue et al., 2011) IP3-receptor inhibition with 2-Aminoethoxydiphenyl borate (2-APB) or Xestospongin-C (XeC) had little or no significant effect on the striated muscle mediated PMTR in chick iris. Consistent with the regulation of skeletal muscle SR stores by Ryanodine receptors (RyRs), however, Ryanodine reduced the PMTR by 42%. Taken together, these findings indicate that 360 < λ < 550 nm light activates CRY signaling to trigger PMT in chick iris striated sphincter muscle by a process involving VGCC mediated Ca^2+^ influx causing Ca^2+^ release from RyR regulated SR stores to initiate muscle contraction.

### PMT in Myotubes

**FIGURE 3:**
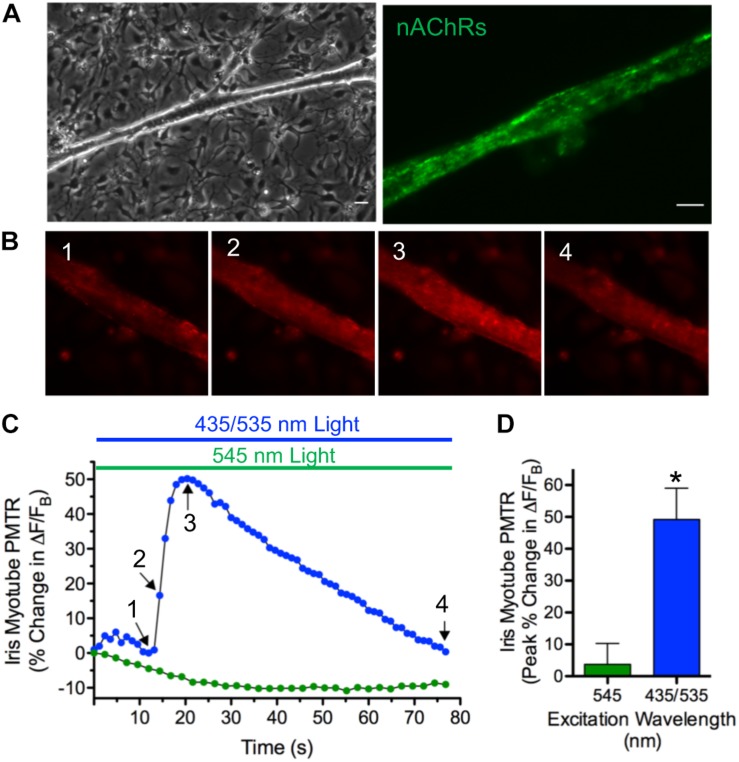
PMT in iris sphincter myotubes*.* **(A)** Iris sphincter myotubes grown in dissociated cell culture display the elongated columnar appearance (left) and diffuse pattern of nAChR puncta expression revealed by AF488-αBgt labeling (right) expected for developing chick striated muscle fibers (Vogel et al., 1972). Scale bars represent 20 μm (left) and 10 μm (right). **(B–D)** Consistent with the CRY photoactivation spectrum in whole iris (Tu, 2004) the iris myotube PMTR is induced by 435 nm light. **(B)** Images 1–4 depict Ca^2+^ fluorescence changes mediated by Rhod-2 in a single myotube exposed to 435 nm and 535 nm (435/535 nm) dual excitation light (at 1.5 and 1.8 × 10^16^ photons s^–1^ cm^–2^, respectively to excite CRY and Rhod-2) before (1) and at start (2), peak (3) and end (4) of the PMTR. **(C)** Specific iris myotube Ca^2+^ fluorescence changes expressed as ΔF/F_B_ in response to 80 sec exposure to 435/535 nm dual excitation light (blue bar and circles; Arrows 1-4 correspond to images 1–4 in panel **(B)** and lack of response after exposure to 545 nm single wavelength light (at 3.0 × 10^16^ photons s^–1^ cm^–2^ green bar and circles). Points reflect % change in ΔF/F_B_ values relative to the value at light onset. Sixteen of 18 iris myotubes tested (89%) displayed PMTRs in response to 435/535 nm dual excitation light. **(D)** Cumulative iris myotube PMTR expressed as peak% change in ΔF/F_B_ (± SEM) in response to dual excitation 435/535 nm light (blue bar; 49 ± 9%, *n* = 16; *N* = 6) or single wavelength 545 nm light (green bar; 4 ± 7%, *n* = 10; *N* = 4).

Cell culture, photo-stimulation and Ca^2+^ imaging approaches were developed to reveal the cellular basis and mechanisms underlying PMTRs. Multinucleated myotubes, resulting from myoblast fusion, first appeared in dissociated cell cultures prepared from E13-14 irises after 3–4 days. Iris myotubes displayed the long cylindrical shape and diffuse expression pattern of nAChR puncta characteristic of chick striated muscle fibers (Vogel et al., 1972; Figure 3A). Consistent with the expression of *CRY1* and *CRY2* transcripts in chick iris and in accord with the action spectrum for its peak CRY-dependent response (Figure 3A) and (Tu, 2004) 435 nm excitation light evoked PMTRs in single iris myotubes (Figures 3B–D). After loading with the fluorescent Ca^2+^ indicator Rhod-2, nearly all myotubes responded to dual excitation 435 and 535 nm (435/535 nm) light, respectively exciting CRY and Rhod-2 with increases in peak Ca^2+^ ΔF/F_B_ of 49 ± 9% that were accompanied by concentric contractions (12 ± 7% shortening) in 5 of the 16 responsive myotubes. The observation that a minor fraction of Ca^2+^ responsive myotubes displayed contractions was correlated with their intermittently loose attachment to the collagen/poly-d-lysine substrate, thereby allowing visual detection of fiber shortening. Presumably this method was insufficient for more reliable contraction detection because in most cases myotubes were uniformly adhered to the substrate. Consistent with CRY activation, the iris myotube PMT required 435 nm light excitation because application of single wavelength 545 nm green light, appropriate for Rhod-2 excitation alone, failed to increase myotube Ca^2+^ fluorescence or induce contractions. These results demonstrate that whole iris PMT is recapitulated in its striated muscle derived myotubes. They further indicate that iris muscle fibers contain a light sensor that, by extension, likely underlies PMT in the whole tissue.

**FIGURE 4:**
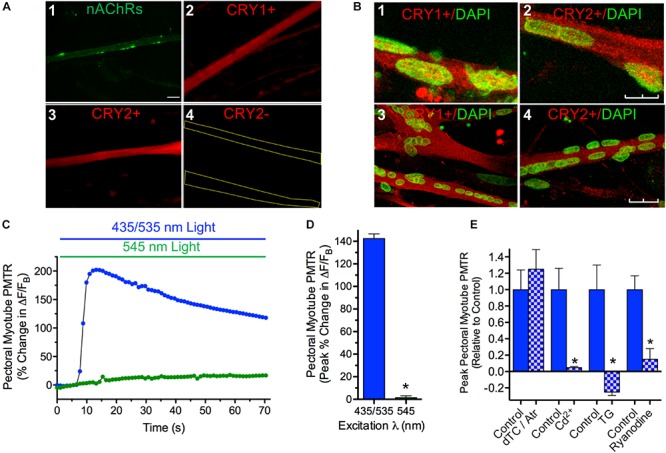
Cytosolic CRY expression in pectoral and iris myotubes; PMT in pectoral myotubes. **(A)** Striated pectoral myotubes in culture identified by morphology and the presence of nAChR clusters (1) express cytosolic CRY1 (2) and CRY2 (3) proteins when viewed at 40X with epifluorescence microscopy. As described in Methods, AF488-αBgt was used to label nAChRs (green), and anti-CRY pAbs followed by AF536-IgG used to label CRY1 and CRY2 immunoreactivity (red). The CRY immunolabeling was specific since none was seen when primary anti-CRY pAb was omitted (e.g. 4, yellow lines outline myotube borders). Scale bar represents 20 μm and applies to 1–4. **(B)** Laser scanning confocal imaging confirms extensive cytosolic CRY1 and CRY2 localization in cultured myotubes immunolabeled as in panel **A** (red) from both iris (1, 2) and pectoral (3, 4) muscle with minimal CRY localization in DAPI-labeled nuclei (green). Images are Z-series projections from 12 to 15 1 μm thick optical sections. Scale bars represents 10 μm in 1 and 2 and 25 μm in 3 and 4. **(C)** Specific pectoral myotube Ca^2+^ fluorescence changes expressed as ΔF/F_B_ in response to 435/535 nm dual excitation light exposure (blue bar and circles) as in Figure 4, and lack of response after exposure to 545 nm single wavelength light (green bar and circles). Points reflect % change in ΔF/F_B_ values relative to the value at light onset. **(D)** Cumulative pectoral myotube PMTRs expressed as peak% change in ΔF/F_B_ (± SEM) in response to dual excitation 435/535 nm light (blue bar; *n* = 74; *N* = 19) or single wavelength 545 nm light (green bar; *n* = 16; *N* = 7). PMTRs from pectoral myotubes were qualitatively similar to those from iris myotubes but were significantly larger (*p* < 0.05; see text comparing PMTRs in Figures 4D,3D). Overall, 98% of pectoral myotubes tested displayed PMTRs in response to 435/535 nm dual excitation light. **(E)** Ca^2+^ dynamics underlying the PMTR in pectoral myotubes. As with whole iris, AChRs are dispensable since peak myotube PMTRs recorded after incubation with 1 μM ATR and 100 μM dTC (15–30 min) were indistinguishable from controls tested in parallel (*n* = 11 and 8, respectively, *p* = 0.5; *N* = 2 for both). As with whole iris, the myotube PMTR requires external Ca^2+^ influx and subsequent release from a Ryanodine sensitive intracellular store. Myotube PMT was reduced by 95 ± 1% in *n* = 4 myotubes incubated for 30 min in RS (normal Ca^2+^) containing 500 μM Cd^2+^ to inhibit influx *via* Ca^2+^ channels when compared with 6 control myotubes (*N* = 2 for both). Incubation in RS containing Thapsigargin (TG, 3uM, 1.5 h) reduced the light response by 125 ± 6% in *n* = 5 test myotubes when compared with 4 time-matched control myotubes (*N* = 2 for both). RyR mediated Ca^2+^ release appeared critical since Ryanodine (100 μM, 1.5 h) significantly reduced PMTRs by 85 ± 13% in *n* = 7 test myotubes when compared with seven time-matched control myotubes (*N* = 2 for both). Results are expressed as mean peak PMTR (± SEM) for myotubes in test RS conditions (blue/white check columns) relative to control myotubes (blue columns) from the same experiments assayed in normal RS.

In order to determine if iris myotube PMT is confined to iris muscle, identical tests were performed using myotube cultures derived from pectoral muscle (Figure 4). Like those from iris, pectoral myotubes had the shape and nAChR distribution pattern characteristic of striated muscle fibers (Figure 4A1). In addition, when viewed with epifluorescence optics pectoral myotubes expressed specific cytosolic CRY1 and CRY2 immunolabeling (Figures 4A2–4) and similar results were obtained for myotubes from iris muscle cultures (data not shown). Confocal images acquired from iris and pectoral muscle cultures co-labeled with anti-CRY pAbs and DAPI (to identify muscle nuclei) confirmed extensive CRY1 and CRY2 cytosolic localization in myotubes with little or no CRY localization detectable in their nuclei (Figures 4B1–4). Because cytosolic CRYs have been linked to light sensitivity in avians (Mouritsen et al., 2004; Wang et al., 2018) it was expected that pectoral myotubes would also exhibit intrinsic photosensitivity. In accord with this expectation, 435/535 nm dual excitation light evoked PMT in pectoral myotubes (Figures 4C,D) featuring peak increases in Ca^2+^ ΔF/F_B_ that were even larger than those from iris myotubes (140 ± 14% versus 49 ± 9%, respectively, *p* < 0.05) while displaying a similar degree and incidence of contractions (11 ± 2% shortening, in 20 of 74 myotubes). As with iris striated myotubes, application of single wavelength 545 nm green light appropriate for Rhod-2 excitation alone failed to increase Ca^2+^ fluorescence or induce contractions in pectoral myotubes. Pharmacological tests were conducted to elucidate mechanisms underlying pectoral myotube PMT (Figure 4E). As with whole irises, peak Ca^2+^ ΔF/F_B_ increases to dual excitation wavelength 435/535 nm light were not significantly different in pectoral myotubes where AChRs were inhibited with the ATR/dTC cocktail (124 ± 23%) compared to controls (88 ± 24%), but were dramatically reduced (by 95%) after blocking Ca^2+^ influx with Cd^2+^. In control experiments using single wavelength 545 nm light, the ATR/dTC cocktail blocked myotube Ca^2+^ responses to AChR activation induced by focal application of RS containing carbachol (CCh, 1 mM) and Cd^2+^ blocked Ca^2+^ responses induced by application of RS containing elevated K^+^ to trigger depolarization-induced Ca^2+^ entry (data not shown). Similar to whole iris, the pectoral myotube PMTR required mobilization of intracellular Ca^2+^ from a SR store because responses to dual excitation wavelength 435/535 nm light were blocked or reduced by 85% following incubations with Thapsigargin or Ryanodine, respectively, relative to untreated controls. These findings demonstrate that like iris (Figure 1) pectoral myotubes display AChR-independent PMT involving release of intracellular Ca^2+^ initiated by 435 nm light-driven Ca^2+^ influx and subsequent Ca^2+^ release from a Ryanodine sensitive intracellular SR store. These results further indicate that cell autonomous PMT involving canonical mechanisms of Ca^2+^ mobilization is a general feature of developing striated muscle.

### Cryptochromes 1 and 2 Mediate Myotube PMT

**FIGURE 5:**
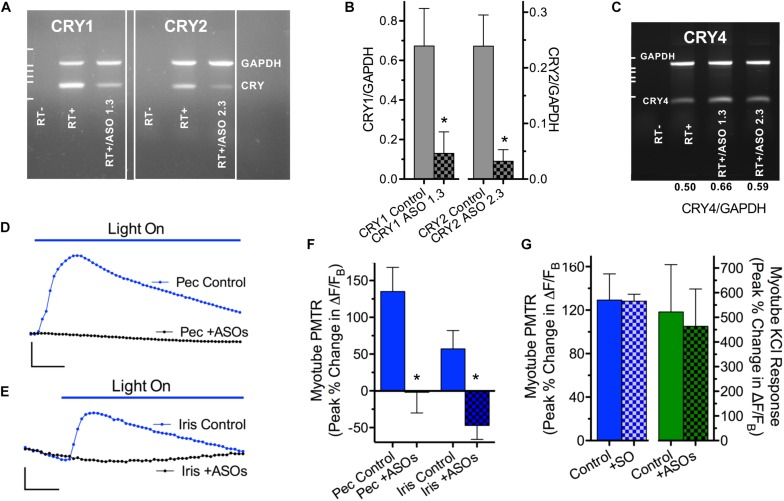
CRY1/CRY2 transcript knockdown prevents myotube PMTRs. **(A,B)** *CRY1* and *CRY2* transcripts assessed by semi-quantitative RT-PCR were reduced in myotube cultures treated with CRY1/CRY2 -specific ASOs. **(A)** Agarose gel depicts *CRY1/GAPDH* and *CRY2/GAPDH* PCR products from co-amplifications with *Gallus*-specific CRY and GAPDH (Set 2) primers using cDNA templates reverse-transcribed from RNA derived from control and ASO-treated pectoral myotube cultures. The ASOs depicted are *CRY1* ASO 1.3 (for *CRY1*) and *CRY2* ASO 2.3 (for *CRY2*). Their sequences are: *CRY1* ASO 1.3 5′-T_1721_GTCTGACCA TCACCAGTTCC_1701_-3′ and *CRY2* ASO 2.3 5′-T_1424_AGTCCACTCCAATGATGCAC_1404_-3′. Respective RT + and RT- designations indicate the presence and absence of cDNA templates generated by reactions containing (+) and lacking (−) RT enzyme. Note that the EtBr fluorescent intensities of *CRY1* and *CRY2* reaction products relative to *GAPDH* are visibly lower for the ASO 1.3 and 2.3 treatment conditions. **(B)** Quantification of *CRY* transcript knockdown. *CRY1 and CRY2* relative to *GAPDH* reaction product intensities were calculated as described in section “Materials and Methods” and compared for PCR amplifications from Control and ASO treated pectoral myotube cultures from agarose gels as in panel **A**. The resulting *CRY/GAPDH* product ratios are depicted for Control (gray columns) and ASO treated cultures (gray/black check columns) on the left (CRY1) and right (CRY2) Y-axes. *CRY/GAPDH* product ratios were obtained in *n* = 3–5 PCR reactions from *N* = 2 RNA preparations. **(C)** *CRY4/GAPDH* PCR products amplified with *Gallus*-specific *CRY4* and *GAPDH* (Set 2) primers from cDNA templates derived from the same control and ASO 1.3 and 2.3-treated pectoral myotube cultures as in panel A1. Note that the EtBr fluorescent reaction product intensities of *CRY4* relative to *GAPDH* are not reduced by ASO treatments; they were even slightly higher (*CRY/GAPDH* ratios at bottom). In both panels **(B,C)**, assuming 1:1 conversion of RNA to cDNA, 150 ng of cDNA template were added to all RT + and RT + /ASO reactions. Dashed lines at left indicate pGEM DNA marker bands at 517, 460, 396, 350 and 222 nt. **(D–F)** *CRY* ASOs that knock down *CRY1* and *CRY2* transcripts prevent PMTRs in both pectoral and iris striated myotubes. Pectoral (Pec) and iris (Iris) myotube cultures were incubated without (Control) or with C*RY1* ASO 1.3 and C*RY2* ASO 2.3 (2–5 μM each) (+ASOs) for 25–48 h, washed with RS, loaded with Rhod-2 and myotube PMTRs assessed in RS as in Figures 3, 4. Exemplar recordings of ΔF/F_B_ in response to dual excitation 435/535 nm light for control (blue circles) and ASO-treated (black circles) pectoral **(D)** and iris myotubes **(E)**. Calibration bars represent 40% and 20% changes in ΔF/F_B_ in panels **D** and **E**, respectively, and 10 s in both. **(F)** Quantification of ASO effect on pectoral and iris myotube PMTRs. Pectoral myotube PMTRs (Peak% Change in ΔF/F_B_) were 135 ± 33% for controls (*n* = 11, blue columns) *versus* −1 ± 27% for ASO treated myotubes (*n* = 13, blue/white check columns) tested in parallel (*N* = 2 for both). For iris myotubes, PMTRs were 57 ± 21% for controls (*n* = 6, blue columns) *versus* −47 ± 19% for ASO treated myotubes (*n* = 5, blue/white check columns) tested in parallel (*N* = 2 for both). (**G**, left) Unlike *CRY* ASOs, a scrambled oligonucleotide (SO) failed to prevent PMTRs. Pectoral (Pec) myotube cultures were incubated without (Control) or with SO (5 μM, + SO) for 25 h and PMTRs. (**G**, right) Specificity of *CRY* ASO PMTR block. Pectoral myotube responses to KCl induced membrane depolarization were indistinguishable in control (*n* = 11, green columns) and ASO treated myotubes (*n* = 5, green/black check columns). Myotube cultures were incubated without or with C*RY1* ASO 1.3 and C*RY2* ASO 2.3 (2 μM each) for 25 h, washed with RS, loaded with Rhod-2 and myotube responses to focal depolarization with RS containing 100 mM KCl assessed in RS using single wavelength 545 nm light as described in section “Materials and Methods.”

A cryptochrome requirement for PMT in iris and pectoral myotubes was assessed using synthetic FANA modified ASOs (AUM BioTech, 21 nt each) to knock down *CRY1* and *CRY2* gene transcripts (Figure 5). Myotubes in cultures incubated with any one of the six *CRY* ASOs tested (5 μM; 24–48 h) were visually indistinguishable from controls indicating the *CRY* ASOs did not grossly affect the survival of differentiated myotubes. *CRY1* and *CRY2* primers were used to amplify products by PCR using equivalent amounts of cDNA templates derived from control and ASO-treated pectoral myotube cultures. Semi-quantitative assessment of *CRY* relative to *GAPDH* PCR amplification products revealed no effect on CRY transcripts with four of the six ASOs tested (data not shown) while two, *CRY1* ASO 1.3 and *CRY2* ASO 2.3, dramatically reduced *CRY1* and *CRY2* transcripts by 87 ± 6% (*N* = 4 experiments) and 91 ± 5% (*N* = 3 experiments) respectively, compared to CRY transcript levels in untreated controls (Figures 5A,B). Since avian CRY4 binds FAD, is expressed in striated muscle, and plays a light-dependent role in magnetoreception (Kubo et al., 2006; Watari et al., 2012; Wang et al., 2018) the possibility that *CRY1* ASO 1.3 or *CRY2* ASO 2.3 might knock down *CRY4* transcripts and thereby reduce PMTRs was tested by amplifying *CRY4* from cDNA templates derived from the same control and ASO treated cultures. This possibility seemed unlikely because *Gallus* mRNAs encoding *CRYs* 1 and 2 respectively have only 39 and 36% identity with *CRY4* mRNA, and because neither *CRY1* ASO 1.3 nor *CRY*2 ASO 2.3 is predicted to bind to corresponding sites on *CRY4*. In accord with this expectation, neither ASO 1.3 nor 2.3 treatments decreased the degree of *CRY4* transcripts relative to untreated controls; instead there was a 20–30% *in*crease in *CRY4* transcripts apparent in two experiments (Figure 5C).

The functional consequences of the observed knockdown in *CRY1* and *CRY2* gene transcripts on PMT was next tested using Rhod-2 loaded myotubes from pectoral and iris sphincter muscle cultures as in Figures 3, 4. Because maximal reduction of whole iris PMTRs required knockdown of *both CRY1* and *CRY2* transcripts (Tu, 2004) pectoral and iris myotube cultures were treated with *both CRY1* ASO 1.3 and *CRY2* ASO 2.3 (2–5 μM; 24–48 h). In nearly all ASO treated myotubes, dual excitation wavelength 435/535 nm light induced slow *decreases* in Ca^2+^ ΔF/F_B_, contrasting with the *increases* typical of untreated control myotubes, including those tested in parallel (Figures 5D–F). The loss of PMT following *CRY* ASO treatment was specific in the sense that PMTRs persisted after treatment with a 21-nt scrambled FANA-modified oligonucleotide (SO). Here, dual excitation 435/535 nm light induced peak increases in Ca^2+^ ΔF/F_B_ that were indistinguishable between SO treated pectoral myotubes (128 ± 6%, *n* = 6, *N* = 2) and untreated controls (129 ± 24%, *n* = 12, *N* = 3) (Figure 5G, left). While the *CRY* ASO 1.3 and 2.3 treatment blocked subsequent myotube PMT, the downstream transduction machinery involving membrane depolarization-triggered Ca^2+^ elevation was unaffected. Thus, when pectoral myotubes from cultures treated with *CRY* ASOs 1.3 and 2.3 were observed using single wavelength 545 nm light they displayed robust increases in Ca^2+^ ΔF/F_B_ in response to focal application of RS containing elevated K^+^ (464 ± 151%, *n* = 5, *N* = 2) that were indistinguishable from those of untreated controls (522 ± 192%, *n* = 11, *N* = 2) including those tested in parallel (Figure 5G, right). These results extend the idea that CRYs mediate PMT in chick iris (Tu, 2004; Figure 3) by identifying CRYs selectively expressed in iris sphincter muscle cells as relevant photosensitive molecules. Because CRYs are also expressed in and responsible for PMT in somatic pectoral myotubes, the results further indicate that developing striated muscle fibers, in general, respond to 435 nm light through CRY photoactivation thereby eliciting PMT *via* downstream canonical mechanisms of Ca^2+^ influx and CICR from a ryanodine sensitive SR store.

## Discussion

Initially based on identifying the cells and mechanisms underlying CRY-mediated PMT in *Gallus* iris, these studies reveal that similar CRY-dependent signaling and transduction mechanisms occur in somatic muscle. The correlation of embryonic iris light response size with muscle type appearance (Figure 1) later confirmed in culture (Figure 3) support the conclusion that developing iris striated muscle fibers contain a PMT-relevant light sensor. Circadian OPN4 and CRY proteins have both been implicated as photo-sensitive proteins relevant to PMT (Tu, 2004; Xue et al., 2011). In amphibian and mammalian smooth muscle irises PMT requires OPN4 (Barr, 1989; Xue et al., 2011; Wang et al., 2017) but its cellular origin remains somewhat unclear. While PMT is linked functionally to smooth iris sphincter muscle in mouse, only a tiny fraction (<10%) of iris smooth muscle fibers from *OPN4-Cre;Rosa-Alkaline Phosphatase* or *OPN4-Cre-Rosa-tdTomato* reporter mice were identified as OPN4 positive, and OPN4 immunoreactivity was undetectable in smooth muscle fibers even though it was present in the sphincter muscle region (Wang et al., 2017). Although RNA transcripts encoding both OPN4 and CRY subtypes were detected in chick iris, our pharmacological findings support earlier results (Tu, 2004) that CRYs alone are required for chick iris PMT (Figure 2). The expression of OPN4 transcripts in chick iris suggests melanopsin may be expressed in smooth muscle or other cell components and contribute to the weak PMT seen at early stages of development or to processes unrelated to PMT at later stages.

Myotubes cultured from chick iris sphincter or pectoral muscle generated similar cellular PMTRs (Figures 3, 4) suggesting that canonical mechanisms of striated muscle contraction are recruited for iris PMT. In iris and myotubes these mechanisms involved extracellular Ca^2+^ influx and CICR from a SR, ryanodine-sensitive intracellular store. Our results support the view that VGCCs responding to membrane depolarization provide a pathway for Ca^2+^ influx in both cases. This conclusion is based on the observation that the VGCC inhibitor Cd^2+^ reduced and blocked PMTRs in iris and myotubes, respectively (Figures 2, 4), and that Cd^2+^ blocked myotube responses to membrane depolarization induced by application of elevated K^+^. CRY signaling could induce membrane depolarization *via* co-modulation of a cytoplasmic K^+^ channel redox sensor β-subunit (K_V_β) as has been proposed for phototransduction in *Drosophila* arousal neurons (Fogle et al., 2011, 2015). Although K_V_β subunits are expressed in muscle (Grande et al., 2003) and circadian redox reactions are implicated in regulating excitability (Bothwell and Gillette, 2018) it is not known if similar mechanisms apply for myotube PMT. Other Ca^2+^-permeable plasma membrane channels may also be recruited for PMTRs in iris and striated myotubes. Results with dTC rule out a contribution from nAChRs, and while transient receptor potential channels, implicated in mammalian iris OPN4-mediated PMT (Xue et al., 2011) are plausible candidates, their contributions were not tested. Neither extracellular Ca^2+^ nor membrane depolarization are required for PMT in frog (smooth muscle) iris where GPCR activation of PLC is thought to cause subsequent IP3 mediated Ca^2+^ release from SR (Barr and Alpern, 1963; Kargacin and Detwiler, 1985; Barr, 1989). For PMT in chick iris and striated myotubes, Ca^2+^ influx induced Ca^2+^ release (CICR) over PLC/IP3- mediated signaling seems more likely because CICR is the canonical route for Ca^2+^ release in striated muscle (Endo, 2009) and because the IP3 inhibitors tested had marginal (2-APB) or no effect (XeC) on chick iris PMT. In accord with results from other species tested, PMT in chick iris and myotubes requires Ca^2+^ release from an intracellular SR store as indicated by the ability of Thapsigargin to reduce iris PMTRs and Ryanodine to inhibit PMTRs in iris and block them in myotubes (Figures 3, 5).

Our results reveal a clear requirement for CRYs in mediating PMT in embryonic iris and pectoral *Gallus* muscle (see below) despite the classification of vertebrate, including avian, CRYs as light-insensitive Type II. Classifying vertebrate CRYs as Type II because their real or theoretical interaction with FAD chromophore predicts light-insensitivity is problematic because experiments to do so have drawn mixed conclusions. On the one hand, while results obtained with CRYs isolated from mammalian cells or expressed in heterologous systems are consistent with some FAD binding, the extent appears small (Hoang et al., 2008; Vieira et al., 2012; Xing et al., 2013) and a recent computational/experimental study suggests vertebrate CRYs express amino acids different from those at positions that confer FAD binding in Type I CRYs (Kutta et al., 2017). However, experimental conditions can influence FAD binding (Özgür and Sancar, 2003) and more flexible requirements may prevail for CRY-FAD interactions to produce phototransduction. Noteworthy here is that expression of a human (Type II) CRY transgene in Type I CRY-deficient *Drosophila* rescues light-dependent magnetosensitivity (Foley et al., 2011) that both human and *Drosophila* CRYs expressed in Sf21 cells display photo-conversion in response to blue light (Hoang et al., 2008) and that an avian CRY (CRY4) binds FAD and plays a light-dependent role in magnetoreception (Kubo et al., 2006; Watari et al., 2012; Wang et al., 2018).

Avian CRY1 and CRY2 have structural features consistent with light-sensitivity. Multiple sequence alignment (ClustalW, v1.83) reveals that they share 14 of the 17 amino acids with CRY4 at sites implicated in FAD binding as well as a triad of tryptophans believed to facilitate light-activated intramolecular electron transfer (Kubo et al., 2006; Kutta et al., 2017). Their light-sensitivity was demonstrated here by showing that PMTRs induced by 435 nm light in iris sphincter and pectoral striated myotubes were blocked after knocking down *CRY1* and *CRY2* transcripts (Figure 5) consistent with observations drawn from pharmacological studies in whole iris (Figure 2). Two ASOs each reduced *CRY1* or *CRY2* gene transcripts by ≈90%, without reducing *CRY4* transcripts, and their combined application blocked PMT in iris and pectoral myotubes, doing so without affecting Ca^2+^ mobilization induced by membrane depolarization. While prior studies using siRNAs suggested that CRY1 and CRY2 additively support whole iris PMT (Tu, 2004) the possibility that the two subtypes have differential actions on myotube PMT was not tested. Because avian cytosolic CRYs are linked to light sensitivity (Mouritsen et al., 2004; Wang et al., 2018) the localization of CRY1 and CRY2 to iris and pectoral myotube cytosol (Figure 4) further supports their role in PMT. Taken together our results are consistent with a scheme where photoactivation of cytosolic CRY stimulates canonical mechanisms of contraction in developing striated muscle *via* muscle membrane depolarization (possibly by K^+^ channel modulation) and subsequent influx of extracellular Ca^2+^ (likely *via* VGCCs and possibly other Ca^2+^ permeable channels) thereby inducing Ca^2+^ induced Ca^2+^ release from a RyR controlled SR store to allow actin-myosin filament interaction.

Our findings further reveal that *Gallus* CRY-mediated PMT, rather than being confined to the iris, is a general feature shared by developing avian striated muscle. Might this CRY- and light-dependent PMT be related to widespread CRY dependent transcriptional control of circadian metabolic processes? While scant clues are available, knowing if light generates CRY-mediated Ca^2+^ mobilization in striated muscle from other species and/or in other cell types may provide useful insights. Given the developmentally transient PMT in developing *Gallus* iris shown here and elsewhere to be CRY-mediated (Meriney and Pilar, 1987; Pilar et al., 1987; Tu, 2004) some crossover functions seem likely since CRY2 and CRY associated redox rhythms have recently been implicated in myogenesis (Grande et al., 2003; Bothwell and Gillette, 2018; Lowe et al., 2018). In this regard it would be useful to know if altering CRY expression and light exposure in myoblasts influences their subsequent differentiation.

## Data Availability Statement

The datasets generated for this study are available on request to the corresponding author.

## Author Contributions

JM conducted most experiments, analyzed and interpreted all results, and prepared the manuscript. MH acquired confocal images, consulted on approaches and result interpretation, and participated in manuscript revisions.

## Conflict of Interest

The authors declare that the research was conducted in the absence of any commercial or financial relationships that could be construed as a potential conflict of interest.

## Footnotes

1 https://grants.nih.gov/grants/olaw/references/ilar91.htm
